# Emerging mechanisms progress of colorectal cancer liver metastasis

**DOI:** 10.3389/fendo.2022.1081585

**Published:** 2022-12-08

**Authors:** Wenhu Zhao, Shipeng Dai, Lei Yue, Fan Xu, Jian Gu, Xinzheng Dai, Xiaofeng Qian

**Affiliations:** Hepatobiliary Center, First Affiliated Hospital of Nanjing Medical University, Nanjing, Jiangsu, China

**Keywords:** colorectal cancer, liver metastasis, cancer stem cells, EMT, tumor micro environment

## Abstract

Colorectal cancer (CRC) is the third most common malignancy and the second most common cause of cancer-related mortality worldwide. A total of 20% of CRC patients present with distant metastasis. The hepatic portal venous system, responsible for collecting most intestinal blood, makes the liver the most common site of CRC metastasis. The formation of liver metastases from colorectal cancer is a long and complex process. It involves the maintenance of primary tumors, vasculature invasion, distant colonization, and metastasis formation. In this review, we serve on how the CRC cells acquire stemness, invade the vascular, and colonize the liver. In addition, we highlight how the resident cells of the liver and immune cells interact with CRC cells. We also discuss the current immunotherapy approaches and challenges we face, and finally, we look forward to finding new therapeutic targets based on novel sequencing technologies.

## Introduction

Colorectal cancer (CRC) is raising more and more attention worldwide because it has become the third most common cancer, with a high incidence of 10%. Meanwhile, CRC accounts for 9.4% of all cancer-related death, the second highest mortality rate among all cancers. China, home to one-fifth of the world’s population, with social development and an aging population, is facing unprecedented cancer prevention and treatment challenges. In China, new cases and deaths of colorectal cancer account for 49.3% and 58.3% of the world’s total, respectively, according to Global Cancer Statistics 2020 (1). The five-year relative survival of colorectal cancer in China is approximately 57%, for metastatic CRC is still low, remaining at around 11% (2). In a retrospective analysis of 780 patients with CRC and liver-only metastases, the median overall survival was 22.8 months. It can be seen that the prognosis of CRC varies widely with or without distant metastasis (3). The hepatic portal venous system, responsible for collecting the majority of intestinal mesenteric drainage, makes the liver the most common site of CRC metastasis. Approximately 15% of patients had already developed liver metastases at the diagnosis, and about 50% of CRC patients will develop liver metastasis during their lifetime (4). However, in many studies on the mechanism of colorectal cancer liver metastasis (CRCLM), the specific mechanism of CRCLM is still unclarified. Clarifying the specific mechanism of CRCLM can provide powerful help for the prevention and treatment of CRCLM.

## Cancer stem cells

CRC is widely considered to be caused by mutations in target oncogenes, tumor suppressor genes, and genes associated with DNA repair mechanisms. Typically, it takes more than a decade to complete successive mutations in multiple genes, such as APC, TP53, TGFBR2, SMAD4, PTEN, and RAS (5). When the original tumor is formed, how it proliferates, invades, and metastases have always been a hot topic in medical research. There are two hypothesized models for continued tumor growth: the traditional and CSC models. The former hypothesis is that every cell in the tumor population can proliferate and differentiate, thus contributing to tumor growth. While the other proposes that only a small group of cells have the potential for tumor proliferation (6).

Cancer stem cells (CSCs) are cells with the ability to self-renew, allowing the cells to generate differentiated cells. These cells are also associated with cancer cell growth and metastasis. Cancer metastasis is related to CSCs, which migrate to distant organs and form metastatic foci. Studies have observed that in the early stages of metastasis, cancer cells show similar gene expression patterns to normal stem cells (7). However, the origin of CSCs has not been clearly understood.

**Figure 1:**
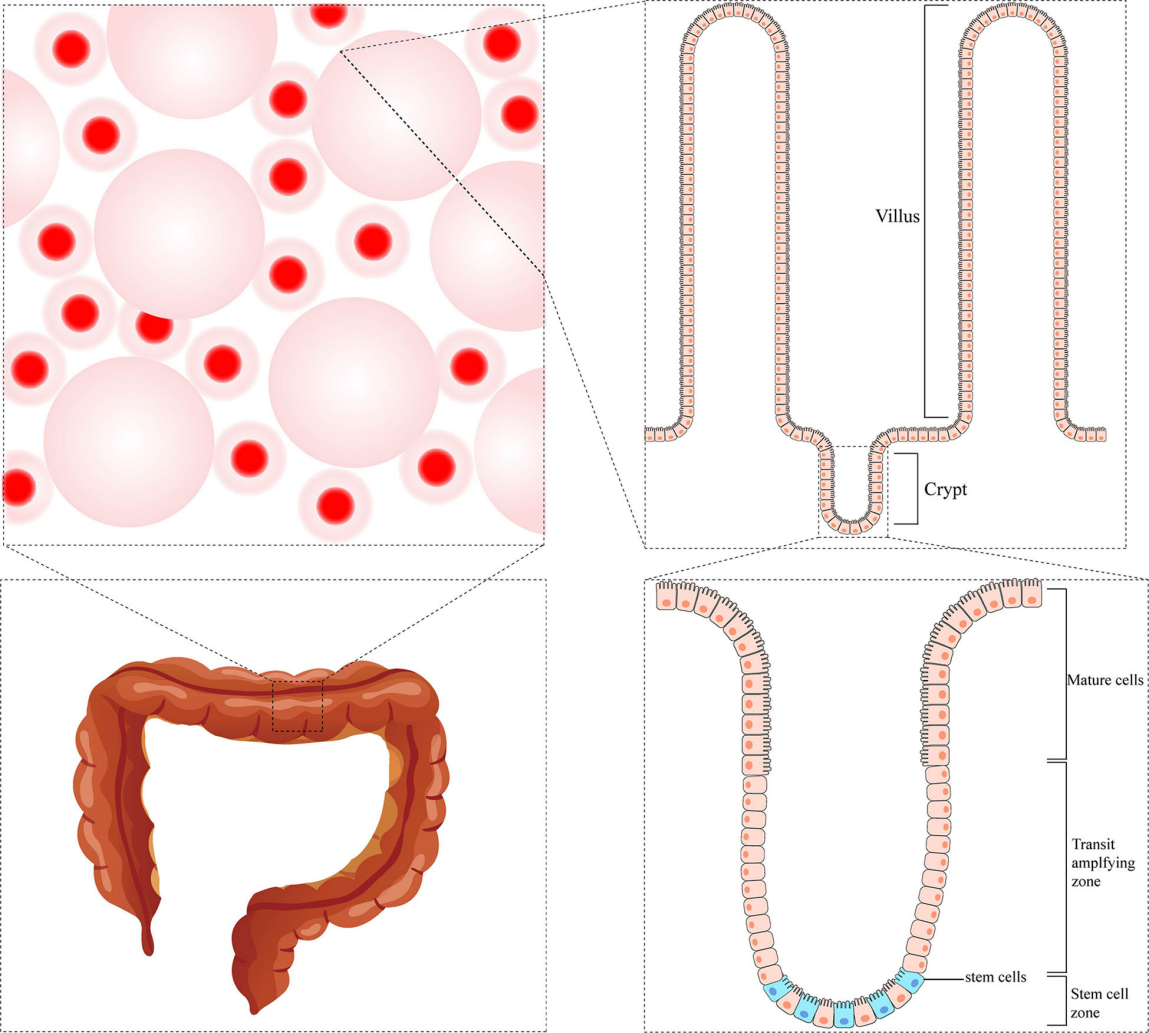
The microstructure of colonic crypt.

So far, most studies have focused on stem cells from the small intestine; paradoxically, stem cells in the colon are much less characterized. The standard physiological structure has many finger-like protrusions in the intestinal lumen. The space between adjacent protrusions is called the crypts of Lieberkühn, which is considered the functional unit of the intestine (8). At the base of each crypt, there is a cell line that can continuously renew epithelial cells called Crypt Base Columnar Cells (expressing high levels of LGR5). Besides, there is another cell line called +4 cells (characterized by prevalent expression of BMI1, HOPX, TERT, and LRIG1). These two populations of cells are thought to be the actual intestinal stem cells that give rise to the epithelial lineages. The two cell populations were initially thought to represent different pools of stem cells with different functional activities, although they can switch between them in both directions. Proliferating LGR5+ cells are thought to be responsible for intestinal homeostasis, which can divide asymmetrically, giving rise to identical daughter cells and transit-amplifying cells that proliferate and differentiate into enterocytes, goblet cells, and endocrine cells during their upward movement through the crypt. In contrast, static BMI1+ cells are considered a reserve pool of stem cells capable of regenerating LGR5+ populations (9). There are differences in crypt structure and cell composition in the colon compared to the small intestine. The colonic crypt does not contain Paneth cells, +4 cells, or BMI1+ cells. Colon stem cells are characterized by LGR5+ (10) or high expression of EphB2 (11). In addition, slow-cycle stem cells were detected in colonic crypts and identified by elevated Notch signaling or LRIG1 expression or DCLK1+ cell subsets (9) (**Figure 1**).

There are two theories of the origin of CRC stem cells: (a) oncogenic mutations accumulating within normal adult cells or ESCs, leading to their uncontrolled proliferation (12); (b) cellular dedifferentiation into a stem-like state, which in a cancer cell would produce CSCs (13). The results of the current studies show that the origin of CSCs may associate with an abnormality of several signaling pathways, which are responsible for controlling the balance between proliferation, differentiation, migration, and renewal in intestinal hemostasis. Here, we list Wnt, Notch, and Hedgehog signaling pathways, which are famous for contributing to the acquisition of cancer stemness.

In the canonical Wnt signaling pathway, the absence of Wnt ligands leads to the degradation of intracellular β-catenin by a destruction complex consisting of Axin, APC, and kinases GSK3β and casein kinase (CK1α) (14). In this case, β-catenin cannot enter the nucleus to initiate transcription of downstream genes. In the presence of secreted Wnt ligands, the canonical pathway is activated. Wnt binds to its Fzd receptors and LRP co-receptors. LRP receptors recruit Dishevelled (Dvl) proteins. The Dvl polymers blocks the phosphorylation of the destructive complex, thereby disrupting its function. This disruption leads to the accumulation and stabilization of the β-catenin, which then transfers into the nucleus (15). There, β-catenin binds to TEF/LEF proteins to form a complex and initiates downstream gene expression, leading to cellular process changes. One of the hallmarks of stem cells is the ability to maintain long telomeres through the function of the TERT gene. Studies have shown that β-catenin can bind to the TERT gene promoter in the nucleus and directly increase its expression level, which links the Wnt pathway to telomerase activity (16). As mentioned earlier, one type of colon stem cell is characterized by LGR5+. LGR5, encoding an R-spondin (RSPO) receptor, is a target gene of the canonical WNT/β-catenin signaling cascade in quiescent as well as cycling stem cells. Barker et al. found that LGR5+ stem cells with high expression of β-catenin formed stem cell clusters in a few days, microadenomas in 3 weeks, and large adenomas in about one month (17). CD44 and CD133 are representative cell-surface markers of CSCs. CD44 and CD133 are further upregulated by WNT and RSPO signals in LGR5+ cycling stem cells (18). These provides reliable evidence that the Wnt signaling pathway promotes normal stem cells to become cancer stem cells.

Notch receptors are transmembrane proteins containing an intracellular domain (ICN) and an extracellular domain. Once the receptors (Notch-1, Notch-2, Notch-3, and Notch-4) are bound with ligands (Jagged-1, Jagged-2, Delta-like-1, Delta-like-3, and Delta-like-4), the pathway is activated. Notch receptors are cleaved twice, and eventually, the N-intracellular domain forms functional cleavage called NICD (19, 20), which enters the nucleus and collaborates with CSL (CBF-1/Suppressor of hairless/LAG1) to initiate transcription of downstream genes (21). Its downstream products are HES and HEY proteins. HES and HEY dimers are essential in regulating the transcription of key genes related to apoptosis, cell cycle, proliferation, differentiation, and metabolism (22). The HES protein has been shown to possess the ability to suppress the expression of a transcription factor called KLF4 (Kruppel-like factor), which has been proven to inhibit colorectal cancer proliferation when overexpressed (23). In addition, it is proved that Notch expression is 20-30 times higher in CSCs than in normal colorectal cancer cells (24). Sikandar S et al. discovered that Notch is a crucial component of CSCs’ self-renewal. It can also prevent apoptosis by inhibiting cell cycle kinase inhibitor P27 and transcription factor ATOH1. Their research also found that several parts of Notch pathways were upregulated, including downstream protein HES, ligand Jagged-1, and receptor Notch-1. Blocking Notch signaling with shRNAs significantly reduces the level of HES1, and cells are found to differentiate (25). The above evidence indicates that Notch plays a vital role in the formation and maintenance of CSCs, which lead to tumor progression and metastasis.

**Figure 2:**
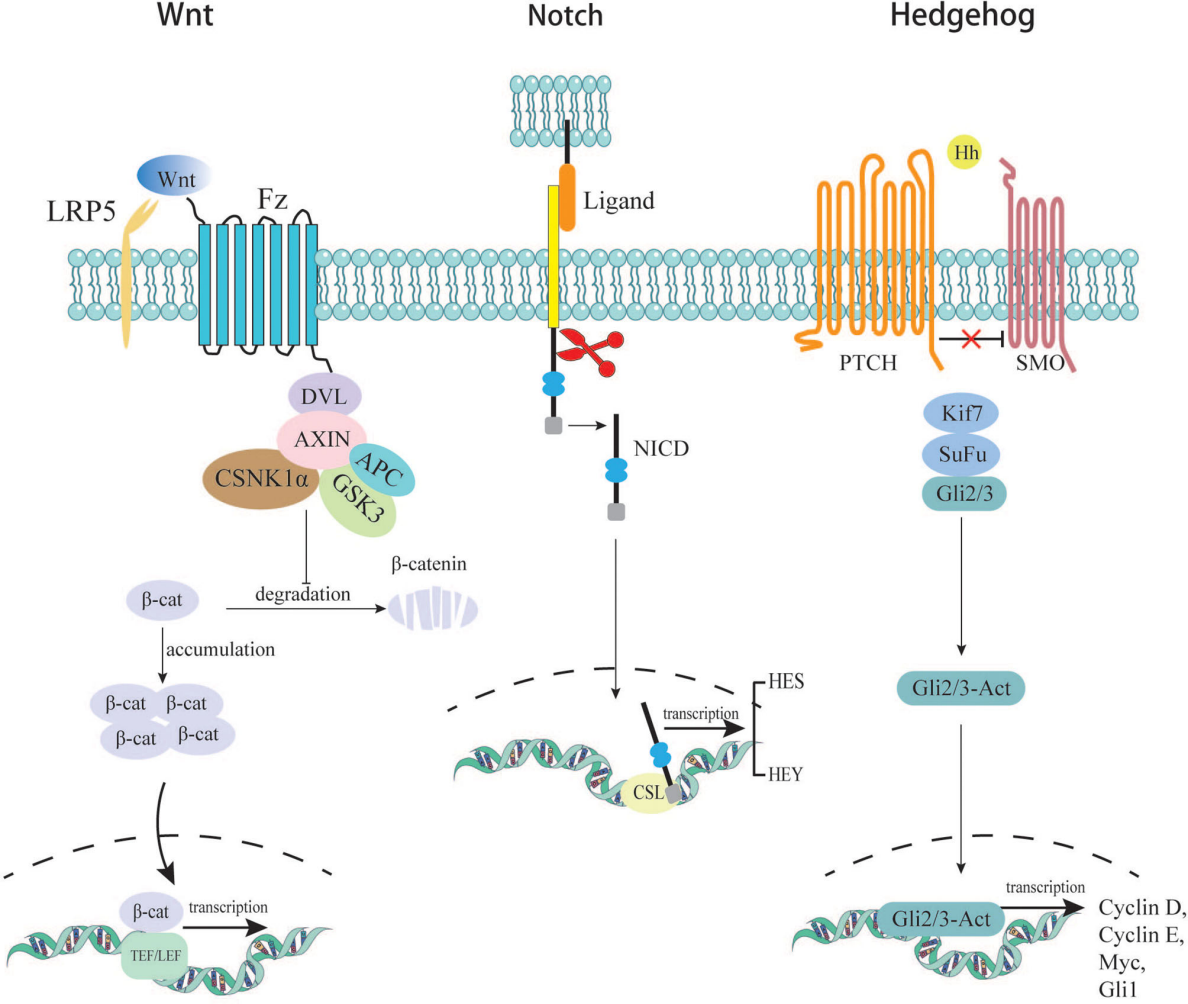
Several signaling pathways related to acquisition of cancer cell stemness.

The Hedgehog (Hh) pathway is a highly conserved signaling system during evolution, which was first discovered in Drosophila melanogaster. Since its discovery, the Hh signaling pathway has been shown to play an essential role in normal embryonic development both in invertebrates and vertebrates (26). In addition, the Hh signaling pathway is also involved in the continuous renewal of intestinal epithelium in adults. The Hh pathway is inactive mainly or active poorly in the adult organism. It will be activated in some specific situations, such as wound healing. Furthermore, the pathway is involved in the maintenance of somatic stem cells and pluripotent cells essential for self-repair and self-renewal in some epithelial tissues. It is suggested that dysregulation of this signaling pathway may be related to the formation of CSCs, thereby promoting the occurrence and development of CRC (27). The specific mechanisms of Hh signaling have been elucidated in previous literature (28). Studies have confirmed that abnormal activation of the Hh pathway is closely related to tumorigenesis in various tissues (29). When Hh pathway is abnormally activated, its downstream genes begin to be transcribed; the downstream products NONAG and OCT4 are important proteins for maintaining the stemness of CRC cells. In the experiment system of Varnat F. et al, the experiment of tracking CD133+ cells allowed them to conclude that human CRC stem cells require active HH-GLI signaling for survival and self-renewal, with increased signaling driving a population expansion in advanced cancers and metastasis (30). Although more than 100 studies on the relationship between Hh and CRC have been conducted, the results are not uniform. Most studies found that the Hh pathway is upregulated in CRC, while about 10% of studies found Hh down-regulated in CRC or unrelated to it (31). Varnat F et al. found that Hh-GLI activity regulates the number of tumorigenic colorectal cancer stem cell populations *in vivo*. Meanwhile, Hh-GLI promotes the proliferation of colorectal cancer cells and is involved in maintaining their ability for self-renewal (30). (**Figure 2**)

## Epithelial-mesenchymal transformation

Growing evidence supports that there is an overlap between EMT stimuli and cancer stem cells, several pathways are shared both in EMT and CSC formation. Song Y, et al. found that Inhibitor Agents whose primary purpose is to inhibit EMT, not only inhibit EMT and metastasis but also suppress the stem cell-like properties (32). Epithelial-mesenchymal transformation (EMT) is the process by which epithelial cells lose their epithelial state and acquire mesenchymal phenotypes. Generally, EMT can occur during embryonic evolution, tissue formation, wound healing, and tissue fibrosis. EMT is associated with tumor initiation, invasion, metastasis, and resistance to therapy in cancer. During EMT, cells lose their epithelial features and, consequently, lose cell-cell and cell-extracellular matrix adhesion. The essential phenotypic change is the loss of epithelial-specific E-cadherin and the acquisition of mesenchymal-specific N-cadherin and vimentin. In this situation, cell polarity is decreased, cell adhesion is weakened, and motility is enhanced, subsequently gaining the ability to invade tissues surrounding the primary tumor, extravasate into lymphatics or blood vessels, travel to distant sites through the circulatory system and lymphatic system, and ultimately colonize a metastatic niche. EMT is a complex process, but it is initiated by a series of EMT-inducing transcriptional factors (EMT-TF) such as Snail, Twist, and ZEB (33, 34).

Snails are a family of transcription factors with zinc finger structure that promotes EMT, mediating invasion and metastasis in many malignant tumors (35). Snail bind specifically to a subset of E-box motifs in target promoters like the E-cadherin promoter, which repress the transcription of E-cadherin, thus inducing the EMT program and promoting tumor metastasis (36). It has been suggested that up-regulation of Snail and transcriptional suppression of E-cadherin may play a crucial role in the progression of CRC (37, 38). Additionally, Snail regulates matrix metalloproteinase (MMP) -2 and -9, proteins that help tumor cells break through the basement membrane and enhance their invasion ability (39). Notably, the Snail-induced EMT effect can make CRC cells exhibit stem cell-like phenotypes. Roy, H. K. et al. found that Snail was overexpressed in 78% of CRC tumor samples than in normal tissues (40). In their experimental system, overexpression of Snail increased the expression of CSC markers CD133 and CD44 (41). Subsequent studies have shown that Snail induces CSC characteristics by regulating the gene encoding interleukin-8 (42).

Slug (also called Snail2) is a member of the Snail family. Snail and Slug are highly homologous in structure (share a similar DNA binding structure of four and five C2H2 zinc finger motifs (ZF), respectively) and involvement of cellular processes (bind to E-cadherin promoter). Evidence showed that the expression of Slug was higher in clinical specimens of colorectal cancer compared to non-cancerous tissues. Overexpression of Slug promoted the EMT progression, downregulating the expression of E-cadherin and upregulating that of vimentin. Specifically, Slug could interact with HDAC6 and then recruited HDAC6 and PRC2 to the promoter of E-cadherin and thus inhibited the expression of E-cadherin, promoting EMT and inducing invasion and metastasis of CRC (43). It is worth mentioning that as the product of the Wnt pathway mentioned above, β-catenin can directly activate Slug (44). In colorectal cancer, the number of isolated tumor cells expressing high levels of nuclear β-catenin at the tumor boundary is strongly associated with metastasis and a low survival rate (45, 46). More interestingly, the characteristic morphologic changes of EMT, which are usually detected at the tumor boundary, are thought to be important in allowing cells to detach, disseminate and eventually metastasize (47, 48). These phenomena suggest a potential relationship between the Wnt pathway and EMT.

The Twist gene, first discovered in drosophila melanogaster, is highly conserved. Twist protein is a basic helix-loop-helix (bHLH) transcription factor. As one of the HLH transcription factors, Twist is mainly expressed in embryos and is involved in differentiating mesenchymal tissue cells such as muscle cells, chondrocytes, and osteoblasts (49). It is also a nuclear transcription factor closely related to tumorigenesis and development, which can induce the EMT process and contribute to the growth, invasion, and metastasis of malignant tumors (50). Twist has the ability to repress epithelial genes like the E‐cadherin encoding gene *CDH1 via* binding to E‐Box motifs in their cognate promoter regions (51). A study showed that upregulation of Twist gene expression in the CRC cell lines induced high expression of vimentin and low expression of E-cadherin, hence promoting the EMT program. More interestingly, CRC cell lines transfected with Twist plasmid showed stronger ability to form liver metastasis (52). In CRC, Twist expression is usually limited to the tumor stroma. It has been suggested that twist-positive cancer cells exist in the stroma of human CRC and these cancer cells have a high mesenchymal phenotype. At the same time, the study compared Twist mRNA levels in the blood of healthy individuals and CRC patients and found that Twist mRNA levels were higher in patients than in healthy individuals (53). These results suggest that Twist has the ability to induce EMT and may be able to predict prognosis by assessing blood mRNA levels. In addition, Twist was found to be involved in regulating the acquisition of stem cell properties by tumor cells and increasing their migration ability (54).

The zinc finger E-box binding homeobox (ZEB)family belongs to the zinc finger protein family and consists of two members: ZEB1 and ZEB2. ZEB proteins simultaneously bind the bilateral zinc finger structure to the E-box sequence to repress the transcription of downstream genes, subsequently decreasing the expression level of E-cadherin (55). High expression of ZEB2 in E-cadherin-positive MDCK cells resulted in significant down-regulation of E-cadherin, loss of cell adhesion, and induction of invasion (56). ZEB performs its subsequent action through a complex formed by binding to CTBP (C-terminal binding protein). The formation of the ZEB/CTBP complex can promote the transcriptional inhibition function of ZEB (57, 58). Experiments have confirmed that ZEB1 can be negatively correlated with E-Cadherin in human colon cancer only when the CTBP expression level is maintained. This result further revealed the possible way ZEB regulates E-cadherin (59). In addition, ZEB can interact with TGF-β (60), Snail (61), and some mi-RNAs (62, 63) to promote the EMT process.

**Figure 3:**
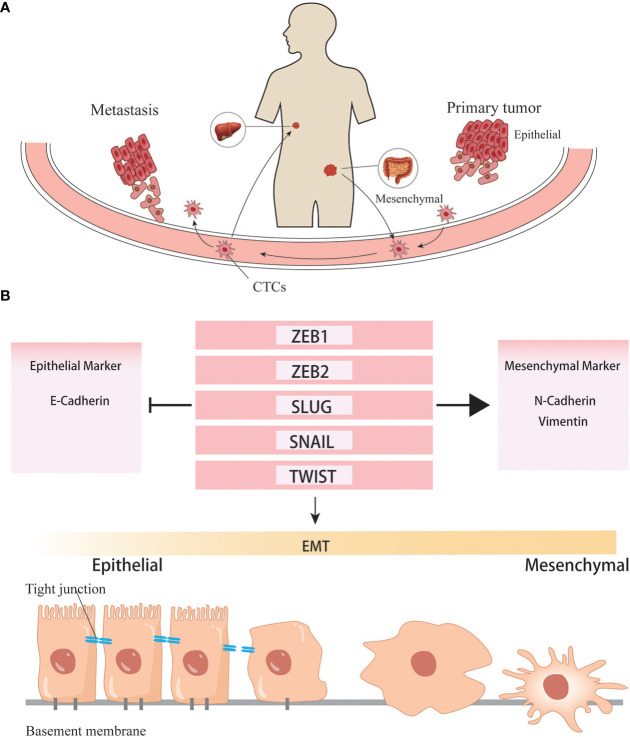
EMT helps the formation of distant metastasis. **(A)** Cancer cells undergone EMT programs acquire the ability to invade into vascular and colonize to distant organ. **(B)** Transcription factors involved in the EMT programes.

As mentioned above, Snail can upregulate CSC markers and promote EMT simultaneously. Activation of the Wnt pathway and high expression of β-catenin not only enable colon epithelial cells to acquire stem cell properties but also promote the initiation of EMT. Meanwhile, Twist also can induce an EMT program and tumor cells to acquire stem-like phenotypes. The evidence above suggests that EMT is orchestrated by a complex network involving regulators of different signaling pathways. Meanwhile, several common pathways may be shared for the induction of EMT and the formation of CSCs. However, there is still a long way to go to elucidate the specific mechanisms (**Figure 3**).

## Circulating tumor cells

**Figure 4:**
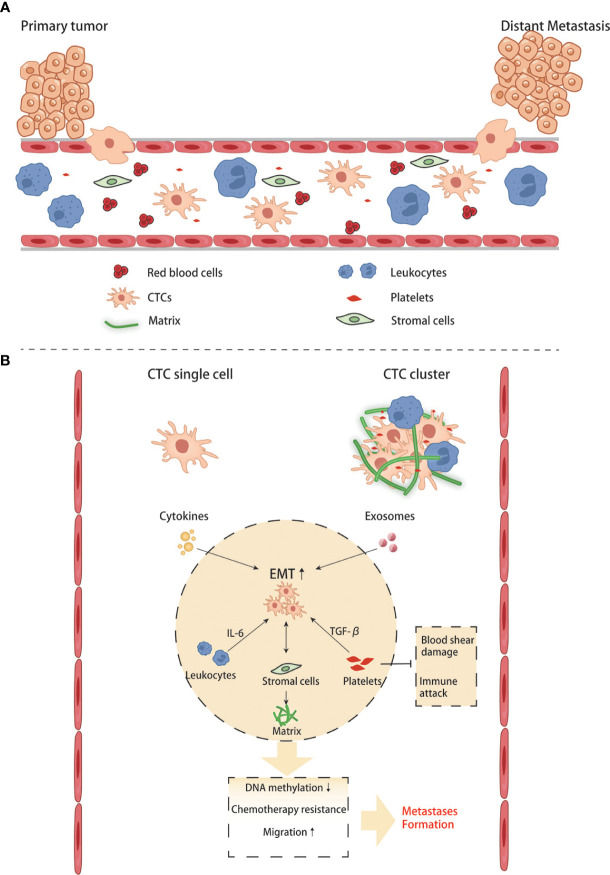
Crosstalk between CTCs and other blood components in the circulation. **(A, B)** CTC single cell and CTC clusters interact with blood cells and stromal cells.

When cells undergo the EMT process, the cell-cell adhesion is significantly reduced, and invasion and migration become easier than cells do not. Under this condition, tumor cells can invade surrounding tissues, intravasate into the blood or lymphatic vessels to reach distant tissues, and complete the process of seeding, proliferating, and forming new colonies. Since most tumor cells disseminate through the blood, these cells traveling in the circulatory system are called circulating tumor cells (CTCs). Some CTCs are shed into the bloodstream from the primary tumor, but they rarely make it to distant organs and form metastases, and are usually killed by immune cells in the bloodstream. Compared with CTCs that have undergone EMT, ordinary CTCs have less ability of invasion, migration, and tumor immune evasion. Only those CTCs that maintain their mesenchymal phenotypes and possess stem cell properties can form new colonies in a suitable metastatic environment. Based on the existing literature, it is generally believed that CTCs exist in the circulatory system as single cells and clusters. The number of clusters was much smaller than that of single cells, but their ability to form metastases was more substantial than that of the latter (64, 65). The high metastasis characteristics of CTC clusters are related to various reasons.On the one hand, the tumor cells within them will undergo phenotypic changes. Studies have found that the DNA methylation level of cancer cells in CTC clusters is significantly lower than that of CTC single cells, especially genes related to stem cell characteristics and cell proliferation (66). Hou et al. found that EMT in CTC clusters was more evident than in single cells (67). A high proportion of mesenchymal cell phenotype was associated with more vital metastasis ability and chemotherapy resistance (68). On the other hand, non-tumor components in clusters also play an essential role: CTC clusters not only contain tumor cells, but also matrix and cell components in the tumor microenvironment, such as platelets, immune cells, and fibroblasts (69). Duda et al. found that CTC clusters contained activated fibroblasts, which could promote the formation of metastatic foci in the mouse lung metastasis model (70). Barbara et al. found that neutrophils contained in CTC clusters could regulate the cell cycle progression of tumor cells, thereby promoting tumor metastasis (71). CD163+ tumor-associated macrophages can secrete IL-6 to activate tumor cells JAK2/STAT3, promoting EMT and CTC-mediated metastasis, which is closely related to poor prognosis of patients (72). The platelets in CTC clusters can secrete TGFβ to activate the TGFβ/Smad and NF-κB pathways to promote the EMT of cancer cells. Meanwhile, they can also cover the surface of tumor cells and form a cloak to resist blood flow shear force and evade immune surveillance (68). CTC is important for two reasons: on the one hand, it can study how tumor cells change during the process between the primary and metastatic organs. On the other hand, it can be applied in clinical practice. Currently, studies have discussed whether CTC can become a necessary standard to judge the prognosis of CRC and guide chemotherapy (**Figure 4**). With technical advancements in the past two decades, methods have become available to investigate the CTCs. Many studies have shown that CTCs are highly valuable for the assessment of the prognosis of both non-metastatic and metastatic CRC. In non-metastatic CRC (nmCRC), the presence of CTCs in blood biopsies can predict liver metastasis (73). Correlation studies have demonstrated that patients with CRC harboring >5 CTC/7.5 ml blood have a worse prognosis with 8-times higher risk of developing metastasis within a year (74).

## Pre-metastatic environment in liver

Metastasis of various malignancies in the liver is influenced by several factors, such as blood flow pattern, tumor stage, and histopathological subtypes. The liver is rich in blood supply, receiving the double supply of the hepatic artery and portal vein. Therefore, gastrointestinal malignancies often metastasize to the liver *via* portal vein circulation. The liver has a unique microenvironment composed of highly specialized resident cells, mainly including hepatic sinusoidal endothelial cells (LSECs), Kupffer cells (KCs), hepatic stellate cells (HSCs), dendritic cells, and resident natural killer cells (75). As circulating tumor cells enter the sinusoidal capillary network through the portal vein system, complex interactions occur between CTCs and these resident cells. According to the existing literature, circulating tumor cells must undergo four overlapping steps to complete seeding and proliferation in the liver: Microvascular phase, pre-angiogenic phase, Angiogenic phase, and Growth phase (76–78).

In the microvascular phase, cells first encountered by CTCs are Kupffer cells and LSECs. Kupffer cells are specialized macrophages in the liver which play a vital part in removing pathogens and induction of local immunity (79). Although Kupffer cells can clear CRC cells in hepatic sinuses by direct phagocytosis and dectin-2 mediated phagocytosis in the early stage of metastasis (80, 81), tumor cells lucky enough to escape immune attack may benefit from it in subsequent colonization and proliferation. In recent years, tumor-derived exosomes have also been shown to promote the formation of liver premetastatic niches for CRCLM by interacting with Kupffer cells. Angiopoietin-like protein-1(ANGPTL-1) can reprogram the secretion pattern of Kupffer cells and enormously reduce the expression of MMP9, thereby preventing leakage of sinusoidal vessels. However, ANGPTL-1 in human CRC exosomes is significantly down-regulated, indicating that down-regulation of ANGPTL1 can affect the liver microenvironment and lead to higher vascular permeability, contributing to the formation of pre-metastatic niche (82). The study of Sun H et al. found that the hypoxia microenvironment in colon cancer tissue can significantly upregulate exosomes containing miR-135a-5p. These exosomes enter the liver with blood circulation. KC phagocytoses them, suppresses immunity, and enhances cell adhesion by affecting LATS2 and CD30, thus promoting liver metastasis of colorectal cancer (83). A recent study demonstrated that Kupffer cells could be recruited by the TCF4-CCL2-CCR2 signaling pathway, which is overexpressed in colorectal cancer and be polarized into M2, which can secrete pro-tumor cytokines such as IL-4, IL-13, VEGF and EGF to promote liver metastasis of colon cancer (84). In addition to those pro-tumor cytokines, TGF-beta1 is quite a remarkable one that contributes to forming a pre-metastatic niche. Kupffer cells can be induced to secret TGF-beta1 through AT1a signaling, thus promoting CRC liver metastasis (85). Yuan N et al. used different antibiotics to control the proportion of intestinal flora in mice to regulate the number of KC in the liver, proving KC’s inhibitory effect on CRC liver metastasis. However, most current studies have demonstrated that KC plays a role in promoting the occurrence of CRC liver metastasis. This contrary conclusion may be caused by different immune components being activated in different experimental systems, or the interaction of other cell populations with KC is ignored (86). LSECs line the sinusoidal vessels with characteristic holes that allow liver cells and HSCs residing in the Disse space to have complete contact with the blood (87). Kupffer cells and LSECs can kill tumor cells by expressing TNF, nitric oxide, and reactive oxygen species (88). The death of tumor cells, tissue damage and inflammatory responses are inevitable, leading to the release of various cytokines, including IL-1, IL-6, IL-8, IL-12, and IL-18, as well as chemokines such as CCL5 (89). These cytokines can recruit more immune cells to enhance local tumor immunity. However, it is worth noting that this local inflammatory response can increase the expression of LSECs adhesion receptors such as E-selectin to enhance the adhesion of cancer cells to endothelial cells, thus increasing the chances of tumor cells entering the Disse space (90). Ou.J. et al. reported that endothelial cells induce EMT of CRC cells by secreting fibronectin extra domain A (EDA), thereby promoting their invasion and metastasis, and revealed that ERK signaling pathway might be A critical pathway mediating this effect (91).

Once tumor cells extravasate from sinusoidal microvasculature into the Disse space, hepatic stellate cells (HSCs) are activated by pro-inflammatory substances in the microvascular phase mentioned above. Hepatic stellate cells are the most abundant non-parenchymal resident cells in the liver, accounting for up to 10% of all resident cells in the liver (92), and their activation can influence the formation of CRC liver metastasis by remodeling the extracellular matrix (ECM) (93). In a recent study, Zhao et al. found that CRC cell‐derived EV miR‐181a‐5p activates HSCs by targeting SOCS3 and activating the IL6/STAT3 signaling pathway in HSCs. In turn, activated HSCs promote liver metastasis by remodeling the liver micro-environment *via* activating the CCL20/CCR6 axis in CRC cells. In this condition, the CRC cells secret more α- SMA and fibronectin (94). Tan et al. demonstrated that interaction between CRC and HSCs could promote the differentiation of HSCs into cancer-associated fibroblasts *via* the CXC4/TGF-β1 axis (95). CAF is a cell population considered to preserve the capability of promoting tumor growth, angiogenesis, and metastasis (96). As a fibroblast, the activated HSC can secrete type I and IV collagen, leading to ECM deposition. It also produces chemokines and cytokines such as CCL2, CCL5, and CCL21 to recruit more inflammatory cells (97).

In each of the above steps, many cytokines and chemokines are produced, which can recruit more inflammatory cells, including peripheral monocytes and macrophages. Studies have shown that macrophages are plastic and can polarize into M1 or M2 with different functions. Generally, M1 macrophages have pro-inflammatory and anti-tumor effects, while M2 macrophages promote tumor growth. M2-mediated tumor growth occurs through the production of growth factors such as VEGF, EGF, FGF2, and TGFβ (98). Zhao S. et al. found that tumor-derived exosomal miR-934 can induce M2 polarization of macrophages *via* the downregulation of PTEN expression and activation of the PI3K/AKT signaling pathway to promote CRLM. This phenomenon reveals a tumor and TAM interaction in the metastatic microenvironment mediated by tumor-derived exosomes that affect CRLM (99). Recruitment, secretion, and re-recruitment formed positive feedback, causing more and more immune cells to accumulate in the local liver parenchyma, resulting in TAM infiltration. TAM secretes matrix metalloproteinases, which degrade extracellular matrix and promote the migration and invasion of tumor cells (98). In addition to macrophages, neutrophils are a group of cells recruited to the liver in the early steps of metastasis. Similarly, recent studies have confirmed that neutrophils can polarize into anti-tumor and pro-tumor types – N1 and N2 (100). Neutrophils can form sticky web-like structures known as neutrophils extracellular traps (NETs) in blood vessels which appear to enhance tumor cell adhesion to sinusoidal vessels. NETs are not cytotoxic to CTCs stuck in hepatic sinuses but enhance their metastatic capacity by enriching tumor interleukin (IL-8), thereby initiating more NET formation and creating positive feedback for liver metastasis (101). Neutrophils cooperate with monocyte macrophages to promote tumor angiogenesis by expressing FGF2, VEGF, and TGFβ (102–104). Angiogenesis is an essential step in tumor growth. For years, the FDA has approved Anti-VEGF and its receptor therapies for treating liver metastases from colorectal cancer. According to Hurwitz, H’s phase III trials, adding Bevacizumab (anti-VEGF agent) to standard chemotherapy improved median survival in patients with metastatic CRC; however, most patients do not respond significantly to this therapy (105). This result may be due to the adverse effects of anti-VEGF drugs on the regular vascular system of the liver, resulting in more fragile and permeable hepatic sinusoidal vessels (106, 107). From the available literature, immune cells (including Kupffer cells, peripheral monocytes, macrophages, and neutrophils) seem to have different or opposite roles at different stages of metastasis formation. This condition involves extremely complex tumor-immune cell or immune cell-immune cell interactions. It has been difficult to precisely control the population of cells that promotes metastasis at any given time.

**Figure 5:**
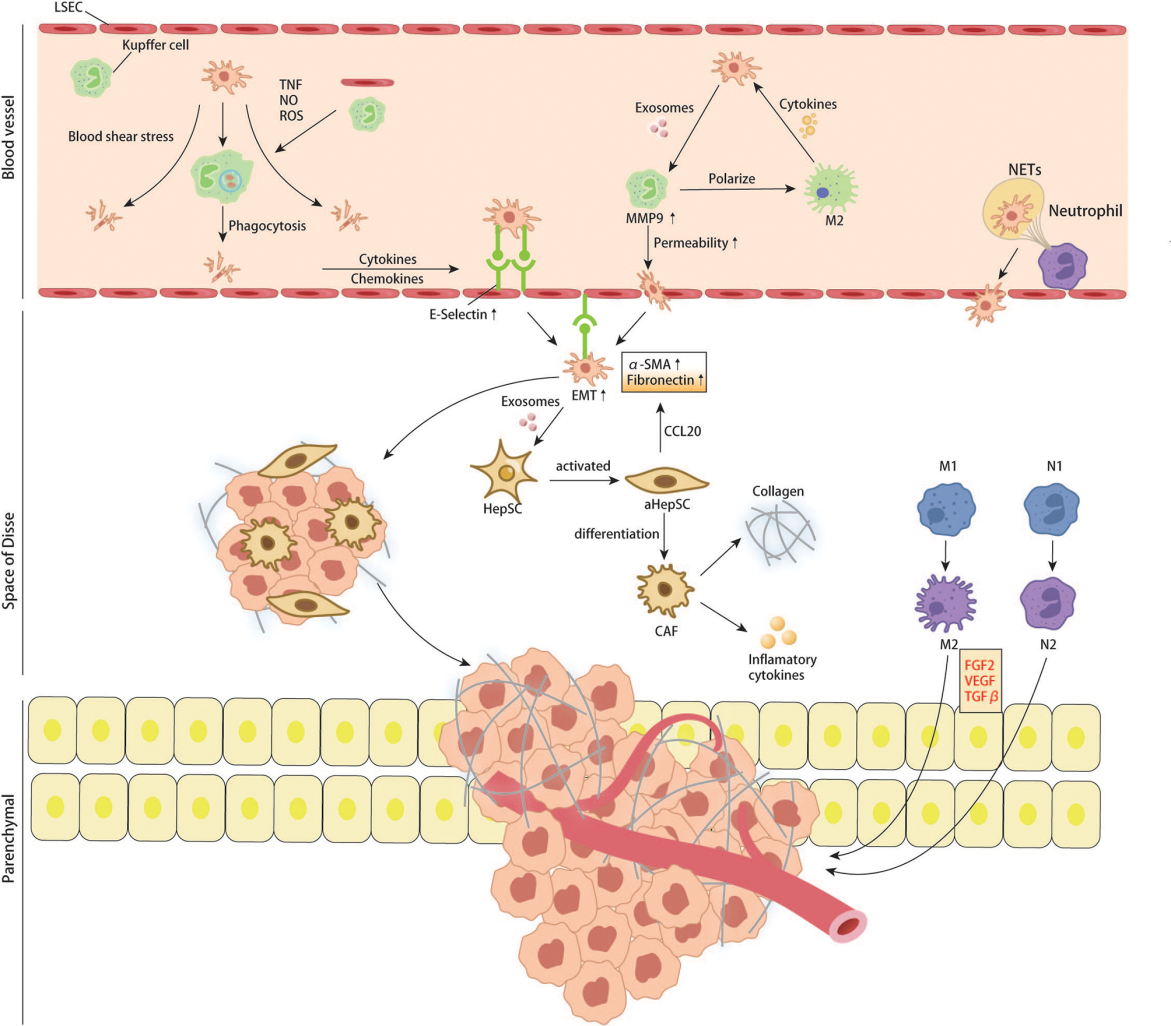
CRC cells interact with resident cells of liver and recruited immune cells in liver sinus, space of Disse and liver parenchymal.

**Figure 6:**
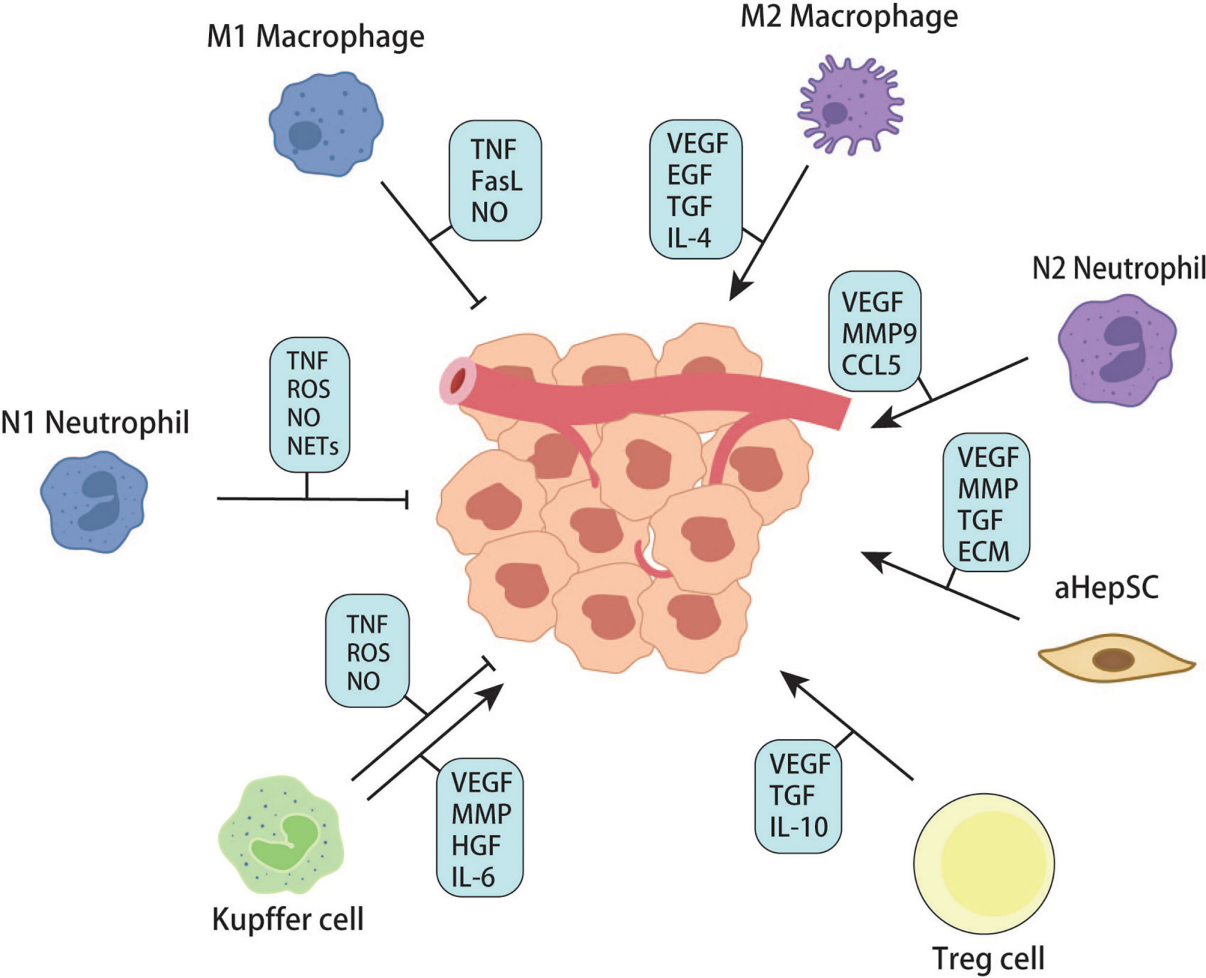
Effects of cells in liver microenvironments on metastatic tumor.

In addition to the effector immune cells mentioned above, regulatory T cells have gradually become the focus of tumor microenvironment research in recent years. Regulatory T cells, featured by CD25 and Foxp3 expression, are immunosuppressive cell populations. Under physiological conditions, Tregs can prevent autoimmunity and regulate immune responses by down-regulating IL-2, producing adenosine, and secreting immunosuppressive cytokines (108). According to the existing literature, Treg infiltration can predict a higher probability of metastasis and poor prognosis in various malignant tumors (109–111). Huang X et al. found that the number of CD4+Foxp3+Treg in the spleen and liver was significantly higher than that in the control group in the murine model of colorectal cancer liver metastasis (112). According to the study of Gu J. et al., in the lactate-rich tumor micro-environment, lactate modulates Treg cell generation and enhances Treg cell function. *In vivo*, the immunomodulatory function of Treg cells decreased after lactate dehydrogenase was used to reduce the concentration of lactate, leading to the enhancement of anti-tumor immunity (113). These studies have directly or indirectly demonstrated the role of Treg in tumor immunity suppression and provided exploration experience for regulating Treg-related tumor immune microenvironments (**Figures 5**, **6**).

Dendritic cells (DCs) are professional antigen-presenting cells that are crucial for the initiation of the immune response to specific antigens through internalization of foreign antigens and subsequent presentation to T cells. The role of liver-derived DCs may be immunosuppressive due to secretion of IL-10 which impedes T cell-mediated cytotoxicity (114). So far, there are few reports on the interaction between dendritic cells and CTC in liver microenvironment, which may be one of the key points to be studied in the future.

Natural killer cell (NK) is an essential component of the liver immune system. Natural killer cells are abundant in the liver and have an important role in resistance to infection and in the clearance of cancer cells. A recent study revealed an interesting mechanism by which tumor cells evade the surveillance of NK cells. Harmon C et al. showed that CRCLM tumors induce NK cells to apoptosis by producing lactate to decrease the intracellular pH of NK cells, resulting in mitochondrial dysfunction (115).

The formation of microenvironment before liver metastasis involves a series of complex interactions and steps. Liver resident cells interact with CRC cells or their exosomes to alter the normal liver microenvironment, including the accumulation of inflammatory mediators, extracellular matrix remodeling, and tumor immunosuppression. These processes overlap with each other, providing a suitable “soil” for the colonization and proliferation of CRC cells in the liver.

## Summary

Colorectal cancer is still the third most common malignant tumor in the world. Liver metastasis is one of the essential factors affecting the mortality of colorectal cancer. According to the “seed-soil” theory, the three critical steps in forming distant metastasis are “cancer stem cells”, “epithelial-mesenchymal transition”, and “tumor microenvironment”. After decades of research, the origin and formation of cancer stem cells have been partially understood, and some critical signaling pathways have been identified to regulate the stemness of cancer cells. Over last decades, people have made efforts to study the origin of cancer stem cells. The hallmarks of CSC in various malignant tumors are identified, which provides us a new method to detect the CSCs and a new tumor therapeutic target.

EMT has been a research hotspot in recent years. Tumor cells undergoing EMT can obtain more vital invasion ability and promote their metastasis to distant areas. Research advances have provided solid evidence for the connection between the activation of the EMT program and the development by carcinoma cells of resistance to therapeutics, not only in the experimental models, but also in clinical settings. Although many EMT-related pathways or star molecules have been identified, much remains obscure about the maintenance of EMT in tumor cells. Many biological properties of cancer cells are determined by non-genetic mechanisms, revealing the limitations of cancer genome sequencing in providing insight into many aspects of cancer cell biology and the need for a combination of complementary approaches such as epigenomics and transcriptomics.

The formation of the microenvironment before tumor metastasis is of great help for tumor colonization and proliferation. In the process of liver metastasis formation of colorectal cancer, these three changes do not occur in sequence but overlap. The liver, which receives blood from the gastrointestinal veins, has the highest rate of colorectal cancer metastasis. The liver has a variety of resident cells, which play various roles in forming the pre-metastasis microenvironment. Some promote tumor adhesion and colonization, some promote tumor proliferation, some regulate the immune microenvironment, and some promote tumor angiogenesis. The tumor immune microenvironment has increasingly become the focus of research. After long-term efforts, people have developed various immunotherapy strategies targeting different cellular or molecular components of the tumor immune microenvironment. For example, anti-PD-1 anti-CTLA-4 therapies target TAMs, TANs, CAFs, and other immune cells. These cells often have both pro-tumor and anti-tumor effects, but the underlying mechanisms are still poorly understood. In the different stages of tumor metastasis, the cell components in the microenvironment often differ significantly in differentiation, function, and cytokine exposure. This change poses a significant challenge for treatment. Although the development of DNA and RNA sequencing technology that pairs tumors and adjacent tissues provides us with new strategies to discover personalized therapeutic targets, it is still necessary to further study the metastatic microenvironment of tumors, and efforts should be made to find more general therapeutic targets.

## Author contributions

WZ, SD and LY were responsible for gathering information of the related research and designing the review. WZ wrote this paper with the help of all participating authors. FX made the figures. JG supported the fund for this review. XQ and XD have contributed to information interpretation, editing and critical revision of the manuscript. All authors contributed to the article and approved the submitted version.
